# Diagnostic performance of the NI-RADS system for detecting recurrence in head and neck squamous cell carcinoma: a monocentric pilot study

**DOI:** 10.1007/s00432-026-06493-x

**Published:** 2026-06-04

**Authors:** Sara Greco, Ilaria Villanova, Nicola Maggialetti, Giovanni Lorusso, Nicola Maria Lucarelli, Chiara Morelli, Claudia Dipalma, Maria Chiara Brunese, Chiara Copelli, Amato Antonio Stabile Ianora

**Affiliations:** 1 Interdisciplinary Department of Medicine, Section of Radiology and Radiation Oncology, University of Bari “Aldo Moro”, 70124 Bari, Italy; 2 Department of Medicine and Health Sciences “V. Tiberio”, University of Molise, 86100 Campobasso, Italy; 3 Interdisciplinary Department of Medicine, Facial Surgery, Operative Unit of Maxillo, University of “Bari Aldo”, University-Hospital “Policlinico Consorziale Di Bari”, Bari, Italy

**Keywords:** Head and neck squamous cell carcinoma, Neck Imaging Reporting and Data System, Surveillance imaging, Magnetic resonance imaging, Computed tomography

## Abstract

**Background:**

This pilot study evaluates the diagnostic performance and clinical relevance of the Neck Imaging Reporting and Data System (NI-RADS) in the post-treatment surveillance of patients with head and neck squamous cell carcinoma (HNSCC), using computed tomography (CT) and magnetic resonance imaging (MRI).

**Methods:**

This retrospective study included 25 patients with histologically confirmed HNSCC treated between August 2021 and December 2024. All patients underwent at least two post-treatment CT or MRI scans, independently reviewed by two radiologists and classified according to the NI-RADS system. Recurrence was confirmed by histopathology or clinical and imaging follow-up. Diagnostic performance metrics, including sensitivity, specificity, positive predictive value (PPV), negative predictive value (NPV), and accuracy, were calculated using a threshold of NI-RADS ≥ 3. Receiver operating characteristic (ROC) analysis was performed for primary and neck sites.

**Results:**

A total of 56 imaging studies and 112 anatomical sites were assessed. Recurrence was confirmed in 36% of primary sites and 27% of neck sites. NI-RADS category 1 showed 0% recurrence, whereas category 4 showed 100% recurrence for both anatomical sites. For the primary site, sensitivity was 80% (95% CI: 56–94%) and specificity 92% (95% CI: 78–98%), with an accuracy of 88% (95% CI: 76–95%). For neck sites, sensitivity was 80% (95% CI: 52–96%) and specificity 98% (95% CI: 87–100%), with an accuracy of 93% (95% CI: 82–98%). The area under the ROC curve was 0.86 for the primary site and 0.88 for the neck site.

**Conclusions:**

NI-RADS showed good diagnostic performance for detecting locoregional recurrence in post-treatment surveillance of HNSCC using CT and MRI. However, given the retrospective design and limited sample size, these findings should be considered preliminary and require confirmation in larger prospective studies.

**Supplementary Information:**

The online version contains supplementary material available at 10.1007/s00432-026-06493-x.

## Background

Head and neck squamous cell carcinoma (HNSCC) represents the seventh most common malignancy worldwide, with an estimated annual incidence exceeding 890,000 new cases and approximately 450,000 deaths, according to GLOBOCAN 2020 data (Sung et al. 2021). Its diagnosis and management pose considerable clinical challenges due to the complex regional anatomy, the functional relevance of involved structures, and frequent presentation at advanced stages. Accurate staging and follow-up are crucial for patient care and rely heavily on multimodal imaging techniques combined with clinical and histopathological assessment (Pynnonen et al. 2017). Imaging plays a pivotal role throughout the care continuum of HNSCC - from initial staging to treatment planning, response evaluation, and long-term surveillance. Cross-sectional imaging modalities such as computed tomography (CT), magnetic resonance imaging (MRI), positron emission tomography/computed tomography (PET/CT), and ultrasound are widely employed, each offering complementary anatomical and functional insights. Despite therapeutic advances, approximately 50–60% of patients experience locoregional recurrence within two years of treatment, and 20–30% develop distant metastases (Denaro et al. 2016). Management typically involves a multidisciplinary approach, with surgery and radiotherapy or chemoradiotherapy as primary modalities, and neoadjuvant chemotherapy used selectively depending on institutional protocols (Machiels et al. 2020). Post-treatment imaging plays a key role in the early detection of recurrence and assessment of therapeutic response. However, interpretation is challenging due to surgery-related changes, fibrosis, inflammation, and anatomical distortion (Chakrabarty et al. 2024). Differentiating between post-therapeutic effects and true tumor recurrence is especially challenging in the early post-treatment phase (< 12 weeks), when false-positive findings due to inflammatory changes are common (Helsen et al. 2017).

While PET/CT offers valuable metabolic information, its specificity is limited in the immediate post-treatment period. Similarly, CT and MRI show variable diagnostic performance depending on tumor site, imaging timing, and reader experience [7–8]. Follow-up protocols typically include clinical examinations by the treating surgeons and imaging studies, in particular CT or MRI, possibly combined with PET/CT (Nekhlyudov et al. 2017).

To improve standardization in the interpretation of post-treatment imaging, the American College of Radiology introduced the Neck Imaging Reporting and Data System (NI-RADS) in 2016 (Aiken et al. 2018). NI-RADS categorizes imaging findings into four risk levels, offering structured follow-up recommendations and enhancing communication across multidisciplinary teams (Aiken and Hudgins 2018). Through the integration of imaging features and suggested clinical actions, NI-RADS aims to improve diagnostic confidence, reduce inter-reader variability, and promote consistency in surveillance strategies.

This pilot study aims to evaluate the diagnostic performance and clinical relevance of the NI-RADS system in a small cohort of HNSCC patients undergoing CT and/or MRI surveillance, by assessing the correlation between NI-RADS categories and clinical-pathological outcomes.

## Methods

### Study population

This retrospective pilot study was conducted on a cohort of patients with HNSCC, treated with curative intent at the Policlinico of Bari between August 2021 and December 2024. Inclusion criteria were: (1) histopathologically confirmed HNSCC; (2) completion of primary treatment (surgery, radiotherapy, chemotherapy, or combinations thereof); (3) availability of at least two post-treatment CT or MRI studies suitable for NI-RADS classification.

**Fig. 1:**
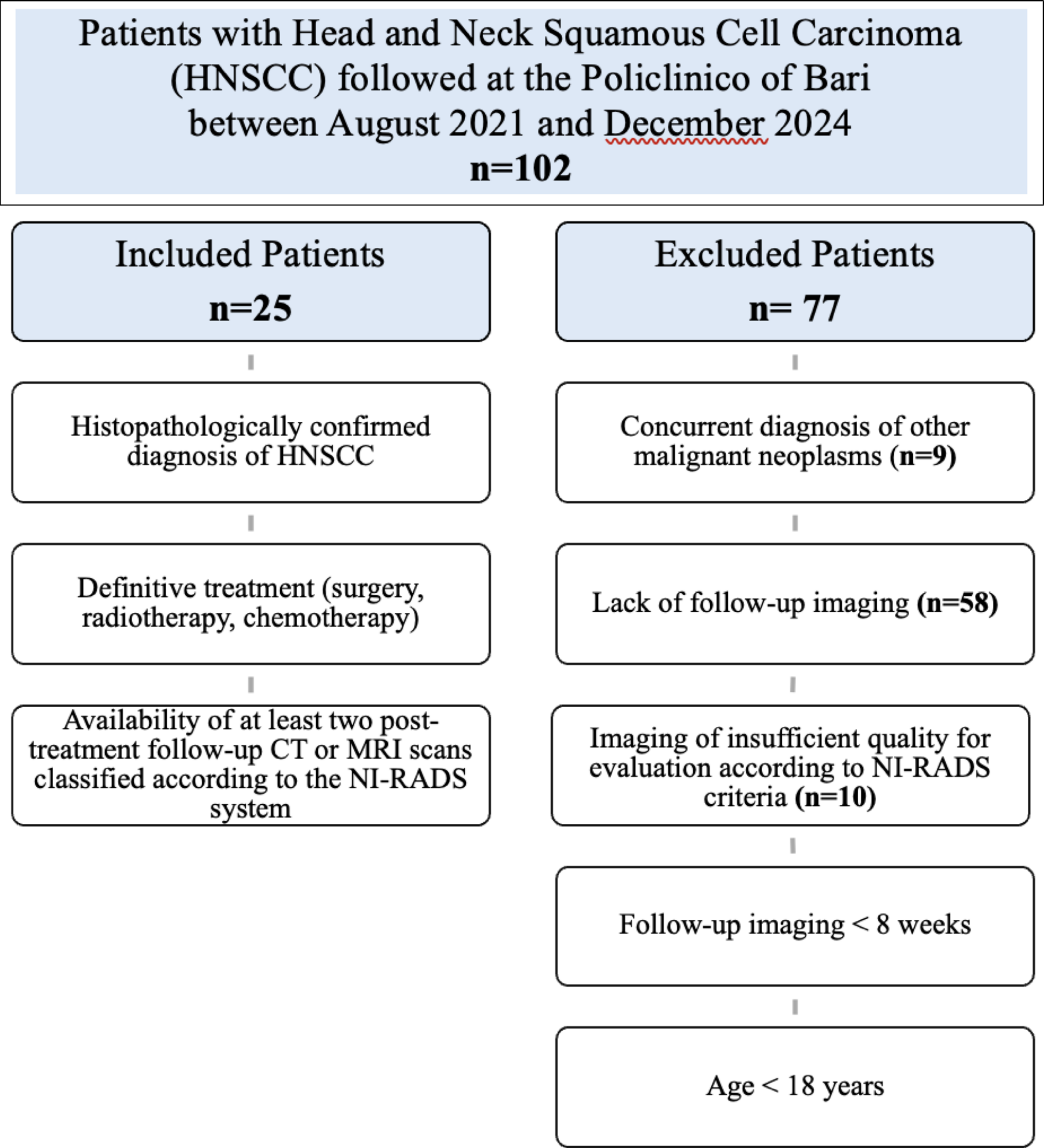
Flowchart of the inclusion and exclusion criteria

Exclusion criteria were: (1) concurrent malignancies; (2) absence of follow-up imaging; (3) inadequate image quality for NI-RADS assessment; (4) follow-up imaging performed less than 8 weeks after treatment completion; (5) age under 18 years (Fig. 1).

Clinical and demographic data were collected from electronic medical records and radiology reports. Histopathological confirmation was considered the reference standard whenever tissue sampling was performed. In cases where histopathological confirmation was not available, recurrence was defined based on unequivocal clinical and radiological progression during follow-up imaging and clinical evaluation. Patients were classified as disease-free if no clinical or radiological evidence of recurrence was observed during follow-up of at least 6 months after the last imaging examination.

This retrospective study was conducted in full accordance with ethical principles, including the World Medical Association Declaration of Helsinki. The study was based exclusively on fully anonymized, pre-existing clinical data, and according to our institutional guidelines, ethical approval was not required. Informed consent was waived due to the retrospective nature of the research.

### CT and MRI protocols

CT scans were performed using a 128-slice multidetector scanner (SOMATOM Definition DS, Siemens Healthineers), with a field of view extending from the external auditory canals to the aortic arch. Scan parameters included: 0.75 mm slice thickness, pitch 1.1, gantry rotation time of 0.33 s, 100 kVp, and 38 mAs. Intravenous contrast (Iomeprol-400; Iomeron, Bracco Imaging SpA, Milan, Italy) was administered at 3 mL/s, with imaging acquisition at 80 s post-injection. Reconstructions were performed at 1.25 mm for axial and 2.5 mm for sagittal and coronal planes.

MRI studies were conducted on a 1.5T scanner (Philips Achieva Nova Dual, Philips Medical Systems, Best, The Netherlands) using a phased-array surface coil. MRI coverage extended from the skull base to the lung apex, including coronal and axial T1-weighted turbo spin-echo, axial and sagittal T2-weighted turbo spin-echo, axial short tau inversion recovery (STIR) T1-weighted, axial fat-suppressed T1-weighted, and 3D contrast-enhanced T1-weighted sequences. Slice thicknesses were 3.5 mm (coronal and sagittal) and 4 mm (axial). Diffusion-weighted imaging (DWI) was acquired with b-values of 0 and 1000 s/mm². A gadolinium-based extracellular contrast agent (Gadovist, Bayer Pharma AG, Berlin, Germany) was administered at 0.1 mL/kg at 2 mL/s, with venous phase imaging performed 120 s post-injection.

All CT and MRI images were archived in the institutional PACS system (Agfa HealthCare).

The choice between CT and MRI during surveillance was not based on a predefined protocol but was determined in routine clinical practice according to multidisciplinary clinical evaluation, tumor location, and imaging availability.

### Image Analysis

All image analyses were performed retrospectively using the institutional RIS system (Agfa HealthCare). Imaging findings were classified according to the NI-RADS criteria to stratify recurrence risk at the primary and neck site (Table 1). Each site was scored from 1 to 4: NI-RADS 1 (no evidence of recurrence), 2 (low suspicion, subdivided into 2a and 2b for the primary site), 3 (high suspicion), and 4 (definite recurrence) (Supplementary Figs. 1 and 2). According to the NI-RADS guidelines, category 2a refers to superficial mucosal abnormalities typically assessed by direct clinical inspection, whereas category 2b refers to deeper ill-defined soft tissue abnormalities generally managed with short-interval imaging follow-up.

**Table 1:** Diagram illustrating the Neck Imaging Reporting and Data System (NI-RADS) categories, including category descriptors, imaging findings, and management recommendations (Aiken et al. 2018)

Category	Category	Imaging Findings	Management
Incomplete	0	New baseline study without any prior imaging available *and* knowledge that prior imaging exists and will become available as comparison*	Assign score in addendum after prior imaging examinations become available
No evidence of recurrence	1	Expected post treatment changes Non-mass-like distortion of soft tissues Low-density “mucoid” mucosal edema No abnormal FDG uptake Diffuse linear mucosal enhancement after radiation	Routine surveillance
Low suspicion	2a	Focal mucosal enhancement, but not mass-like Focal mild to moderate mucosal FDG uptake	Direct visual inspection
	2b	Deep, ill-defined soft tissue, not discrete Little to no differential enhancement Mild or moderate FDG uptake	Short-interval follow-up (3 months), repeat PET
High suspicion	3	New or enlarging primary mass or lymph node Discrete nodule or mass with differential enhancement Intense focal FDG uptake	Image guided or clinical biopsy
Definitive recurrence	4	Pathologically proven or definite radiologic and clinical progression	Clinical management

*FDG* fluorine-18-2-fluoro-2-deoxy-D-glucose, *NI-RADS* neck imaging reporting and data systems

*Morphologically abnormal features that are definitive = new necrosis or gross extra nodal extension as evidenced by invasion of adjacent structures

Evaluated imaging features included lesion size, margins, enhancement pattern, and diffusion restriction on DWI sequences. Special attention was paid to differentiate recurrence from post-treatment changes such as fibrosis or necrosis.

Two radiologists independently reviewed the studies. The radiologists were blinded to clinical outcomes, histopathological results, and follow-up data at the time of image interpretation. Discordant or doubtful cases were resolved by consensus in consultation with a senior radiologist specialized in head and neck imaging.

### Data analysis

Statistical analysis was performed using SPSS version 26.0 (IBM SPSS Statistics, Armonk, NY, USA). Continuous variables are reported as mean ± standard deviation (SD), and categorical variables as absolute values and percentages.

The primary unit of analysis was the anatomical site, including the primary site and neck site, each independently classified according to the NI-RADS system.Diagnostic performance was evaluated by calculating sensitivity, specificity, positive predictive value (PPV), negative predictive value (NPV), and accuracy using a binary classification with NI-RADS ≥ 3 considered positive and NI-RADS 1–2 considered negative. Associations between NI-RADS categories and clinical or pathological outcomes were evaluated using Fisher’s exact test, with significance set at α = 0.05.

Inter-reader agreement was assessed using Cohen’s kappa statistic.

The overall diagnostic performance of NI-RADS was assessed using a receiver operating characteristic (ROC) curve. ROC curves were generated using both a binary threshold (NI-RADS ≥ 3 considered positive) and NI-RADS categories (1–4) as an ordinal predictor. Area under the curve (AUC) was calculated separately for primary site and neck site, with values close to 1.0 indicating excellent discriminative ability and those near 0.5 suggesting no diagnostic value. A NI-RADS threshold ≥ 3 was selected because categories 3 and 4 represent high-risk findings requiring diagnostic or therapeutic intervention, whereas categories 1 and 2 are generally managed with routine or short-interval follow-up.

Given that multiple observations per patient (anatomical sites and repeated imaging examinations) were included in the analysis, statistical tests were performed under the assumption of independence; therefore, results, particularly p-values, should be interpreted with caution.

## Results

The initial cohort included 102 patients with HNSCC treated with curative intent. 58 patients were excluded due to lack of post-treatment follow-up imaging, 10 due to poor image quality unsuitable for NI-RADS classification, and 9 due to the presence of concurrent malignancies. The final sample comprised 25 patients (mean age 65 ± 14 years), composed of 19 males (76%) and 6 females (24%).

Tumor locations were distributed as follows: oral cavity (68%), oropharynx (12%), larynx (12%), and hypopharynx (8%) (Table 2).

**Table 2:** Characteristics of the Study Cohort

Characteristics	General Population (*n* = 25)
Age, mean (standard deviation)	65 (SD ± 14)
Sex	
Women	6/25 (24%)
Men	19/25 (76%)
Tumor location	
Oral cavity	17/25 (68%)
Oropharynx	3/25 (12%)
Larynx	3/25 (12%)
Hypopharynx	2/25 (8%)
Treatment	
Surgery	21 (84%)
Combined treatment (surgery + radiotherapy/chemotherapy)	4 (16%)
Surgical management	
Neck dissection	9/25 (36%)
Median follow-up period	19 months (range 6–22 months)

A total of 56 follow-up imaging studies were analyzed: 37 (66%) MRI and 19 (34%) CT scans, performed between 8 weeks and 22 months post-surgery. Most patients (84%) underwent two follow-up scans, while 8% had three and the other 8% had four.

Each imaging examination was evaluated separately for the primary site and the neck according to the NI-RADS framework, resulting in two evaluated anatomical sites per examination. Overall, 112 anatomical sites (56 primary and 56 neck) were assessed using the NI-RADS system. Recurrence was confirmed in 36% (20/56) of primary sites (15 by histopathology confirmed and 5 by imaging and clinical progression) and in 27% (15/56) of neck sites (11 by histopathology confirmed and 4 by imaging and clinical progression). In 16% (9/56) of cases, recurrence was present at both sites.

At the primary site, NI-RADS 1 lesions (*n* = 28) were confirmed by follow-up in 23 cases and by histopathology in 5 cases. NI-RADS 2a lesions (*n* = 4) were equally confirmed by follow-up (*n* = 2) and histopathology (*n* = 2). Among NI-RADS 3 lesions (*n* = 14), most were confirmed histologically (*n* = 11), while 3 cases were confirmed by follow-up. All NI-RADS 4 lesions (*n* = 5) were histologically confirmed.

At the neck site, NI-RADS 1 lesions (*n* = 36) were confirmed by follow-up in 25 cases and by histopathology in 11 cases. NI-RADS 2 lesions (*n* = 7) included 3 cases confirmed by follow-up and 4 by histopathology. Among NI-RADS 3 lesions (*n* = 11), 7 were histologically confirmed and 4 by follow-up. All NI-RADS 4 lesions (*n* = 2) were confirmed by histopathology.

Recurrence rates varied significantly among NI-RADS categories. Both primary and neck sites showed 0% recurrence in NI-RADS 1 and 100% in NI-RADS 4. For categories 2 and 3, recurrence rates differed between sites: primary site had 55% recurrence (22% for 2a, 33% for 2b), and 79% for category 3; neck site had 43% for category 2 and 90% for category 3 (Table 3).

**Table 3:** Distribution and recurrence rates according to NI-RADS categories for primary site and neck lesions

Category	Distribution by category	Recurrences	Recurrence rate (%)
Primary site			
NI-RADS 1	28 (50%)	0 (0%)	0
NI-RADS 2a	4 (7%)	2 (10%)	50
NI-RADS 2b	5 (9%)	3 (15%)	60
NI-RADS 3	14 (25%)	11 (55%)	79
NI-RADS 4	5 (9%)	5 (25%)	100
**Total**	**56**	**20**	**36**
Neck Site			
NI-RADS 1	36 (64%)	0 (0%)	0
NI-RADS 2	7 (12%)	3 (20%)	43
NI-RADS 3	11 (20%)	10 (67%)	90
NI-RADS 4	2 (4%)	2 (13%)	100
**Total**	**56**	**15**	**27**
Primary and neck site			
NI-RADS 1	64 (57.1%)	0 (0%)	0
NI-RADS 2*	16 (14.3%)	7 (20%)	44
NI-RADS 3	25 (22.3%)	21 (60%)	84
NI-RADS 4	7 (6.3%)	7 (20%)	100
**Total**	**112**	**35**	**31**

NI-RADS showed good predictive performance. For the primary site, sensitivity was 80% (95% CI: 56–94%), specificity 92% (95% CI: 78–98%), positive predictive value 84% (95% CI: 60–97%), negative predictive value 89% (95% CI: 74–97%), and overall accuracy 88% (95% CI: 76–95%). For neck sites, sensitivity was 80% (95% CI: 52–96%), specificity 98% (95% CI: 87–100%), positive predictive value 92% (95% CI: 64–100%), negative predictive value 93% (95% CI: 80–98%), and overall accuracy 93% (95% CI: 82–98%) (Table 4). Fisher’s exact test revealed significant correlation between NI-RADS categories 1 and 3 and clinical-pathological outcomes for both anatomical sites (*p* < 0.05). Category 2 did not significantly correlate with absence of recurrence (*p* > 0.05). Category 4 showed a statistically significant correlation for the primary site (*p* < 0.05; *p* = 0.004), while the result for the neck site was not significant (*p* > 0.05, *p* = 0.06).

**Table 4:** Diagnostic performance of NI-RADS using a threshold ≥ 3 for detecting recurrence

Site	Sensitivity (%)	Specificity (%)	PPV (%)	NPV (%)	Accuracy (%)
Primary site	80	92	84	89	88
Neck site	80	98	92	93	93

NI-RADS categories were dichotomized for diagnostic performance analysis (NI-RADS ≥ 3 considered positive and NI-RADS 1–2 considered negative)

The inter-reader agreement analysis showed a moderate agreement with a Cohen’s kappa of 0.58 and a substantial agreement with a weighted kappa of 0.73, supporting the reproducibility of NI-RADS scoring between readers.

**Fig. 2:**
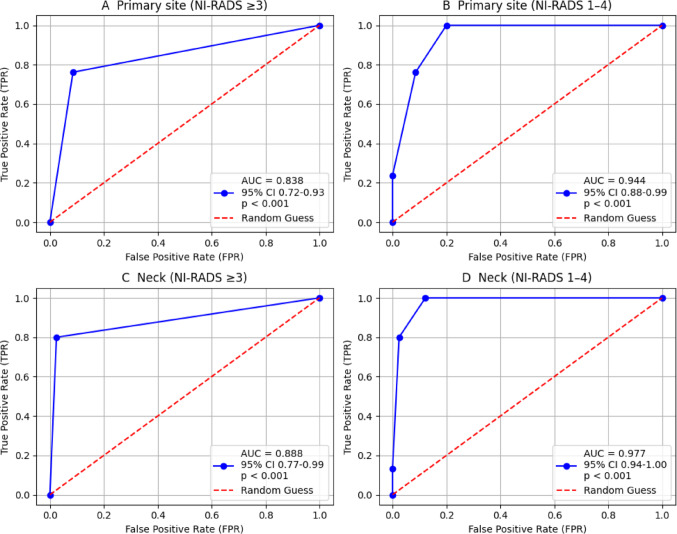
ROC analysis of NI-RADS for detection of locoregional recurrence. **A** ROC curve for the primary site using the clinically relevant threshold NI-RADS ≥ 3. **B** ROC curve for the primary site using NI-RADS categories 1–4 as an ordinal predictor. **C** ROC curve for the neck site using the threshold NI-RADS ≥ 3. **D** ROC curve for the neck site using NI-RADS categories 1–4 as an ordinal predictor. AUC values are shown with corresponding 95% confidence intervals

ROC curve analysis yielded AUC values of 0.86 for the primary site and 0.88 for the neck, confirming the high discriminative ability of NI-RADS (Fig. 2).

## Discussion

This pilot study evaluated the diagnostic performance of the NI-RADS system in the post-treatment surveillance of HNSCC patients, focusing on its correlation with clinical and pathological outcomes. Our preliminary results suggest that NI-RADS may represent a useful and reproducible tool for stratifying recurrence risk in post-treatment surveillance. However, given the exploratory nature of this retrospective pilot study, these findings should be interpreted cautiously and require confirmation in larger prospective cohorts before being translated into clinical decision-making.

NI-RADS were originally designed for use with contrast-enhanced CT, with or without PET, assigns scores from 1 to 4 to the primary and neck sites based on the level of suspicion, offering standardized management recommendations (Krieger et al. 2017). According to ACR guidelines, combined PET/CT at 8–12 weeks post-treatment remains the preferred imaging modality (Aiken and Hudgins 2018). However, contrast-enhanced CT and MRI, while lacking metabolic data, provide high-resolution anatomical detail, and are more accessible. FDG-PET, though valuable, may yield false positives—particularly in NI-RADS category 3—and is often limited by availability and cost (Krieger et al. 2017).

Our results align with those of Li et al.’s meta-analysis including 12 studies and 1,996 lesions reporting an overall NI-RADS specificity of 94% and moderate sensitivity of 69%, with an AUC of 0.90. Specificity was particularly high for both primary (95%) and neck (99%) sites, reinforcing NI-RADS’s utility in ruling out recurrence in negative imaging studies. These findings support the diagnostic value of CT and MRI as practical alternatives to PET/CT in follow-up imaging (Li et al. 2024). While previous meta-analyses have evaluated the overall diagnostic performance of NI-RADS, the present study provides a real-world pilot evaluation of the system in routine post-treatment surveillance using both CT and MRI in a single-center cohort.

In our cohort of 56 post-treatment CT and MRI exams, NI-RADS demonstrated strong diagnostic accuracy. ROC analysis considering NI-RADS as an ordinal scale demonstrated even higher discriminative ability compared with the binary threshold (NI-RADS ≥ 3), confirming the value of NI-RADS as a risk stratification system.

Categories 1 and 3 showed a significant correlation with clinical outcomes, reinforcing their role in identifying low- and high-risk. Category 4 showed 100% recurrence in both primary and neck sites, although statistical significance for neck sites was not achieved due to limited sample size. Conversely, NI-RADS category 2 did not reach statistical significance. This finding should be interpreted cautiously given the limited sample size and number of events. Rather than indicating absence of association, the lack of statistical significance likely reflects limited statistical power in this intermediate-risk category. Importantly, NI-RADS 2 represents an intermediate and clinically indeterminate category, often reflecting post-treatment inflammatory or fibrotic changes that may mimic recurrence. This inherent uncertainty likely contributes to its variable predictive value and supports the need for close imaging or clinical follow-up rather than immediate intervention.

Overall, the progressive increase in recurrence rates across NI-RADS categories supports its interpretation as an ordinal risk stratification system.

This finding is consistent with previous studies and further supports the clinical validity of NI-RADS (Baba et al. 2023).

A recent narrative review by Vertulli et al. highlighted NI-RADS’s diagnostic reliability across imaging modalities but also emphasized challenges related to a high frequency of indeterminate findings—especially in category 2—and inter-reader variability. The authors suggested that advanced techniques such as diffusion-weighted imaging (DWI) and PET/MRI could enhance diagnostic accuracy and reduce variability (Vertulli et al. 2025).

Emerging imaging modalities—such as dual-energy CT, blood oxygen level-dependent MRI, and hybrid PET/MRI—alongside radiomics and machine learning approaches, offer promising avenues to improve tissue characterization and personalized risk stratification (D’Angelo et al. 2017, Chaudhry et al. 2019, Haider et al. 2020, Nougaret et al. 2019, Forastiere et al. 2018). Integration of clinical factors like HPV status and molecular data may further refine recurrence prediction. Despite its structured approach, NI-RADS faces challenges in intermediate-risk categories where benign post-treatment changes mimic malignancy. Ongoing refinement incorporating advanced imaging biomarkers and AI-driven tools is essential to enhance its clinical utility [21–22].

To date few studies have assessed NI-RADS performance across both CT and MRI in a general HNSCC population regardless of primary site or treatment modality. Despite the limited sample size, our findings are consistent with those of Dinkelborg et al. and with recent studies suggesting that specific MRI protocols can improve detection of nodal metastases and extranodal extension. These data support the use of CT and MRI as reliable first-line modalities in surveillance (Dinkelborg et al. 2021, Lorusso et al. 2025, Maggialetti et al. 2025).

This study has several strengths. It provides a real-world evaluation of NI-RADS in routine post-treatment surveillance using both CT and MRI. Imaging studies were independently reviewed by two radiologists, with consensus resolution of discrepancies, supporting reproducibility. In addition, both primary site and neck sites were systematically assessed, allowing a comprehensive evaluation of locoregional recurrence.

However, several limitations should be acknowledged. These include the retrospective nature of the study, small sample size, single-center setting, relatively limited follow-up duration, heterogeneous use of imaging modalities, and the absence of systematic PET/CT as a reference standard. In particular, the relatively short minimum follow-up duration (6 months) may have led to underestimation of late recurrences. This limitation is especially relevant when interpreting the high specificity and negative predictive value observed in our cohort, which may therefore be slightly overestimated. In addition, because multiple imaging examinations and anatomical sites were analyzed for each patient, observations were not fully independent and may represent clustered data.

Furthermore, detailed tumor staging (T and N categories and UICC stage) and surgical data were not consistently available due to the retrospective nature of the study. Finally, this study focused on locoregional recurrence and did not systematically assess distant metastatic disease, which is an important component of HNSCC surveillance.

Given these limitations, the diagnostic performance estimates reported in this pilot study should be interpreted as preliminary and hypothesis-generating. Larger prospective multicenter studies with standardized imaging protocols and longer follow-up are needed to validate these findings and to define the role of NI-RADS in guiding clinical decision-making. Future research should also explore the integration of advanced imaging techniques and artificial intelligence to improve diagnostic accuracy, particularly in indeterminate categories. Continued refinement, and harmonization of imaging protocols will be essential to establish NI-RADS as a robust tool in post-treatment oncologic imaging and to ultimately improve patient outcomes.

## Conclusions

This pilot study suggests that the NI-RADS system may represent a useful tool for structured reporting and risk stratification in post-treatment surveillance of HNSCC using contrast-enhanced CT and MRI. In our cohort, NI-RADS categories showed a meaningful correlation with clinical outcomes, with categories 1 and 3 demonstrating a significant association with recurrence risk, while category 2 remained indeterminate. However, given the retrospective design and the limited cohort size, these findings should be considered preliminary and hypothesis-generating. Larger prospective studies are required to confirm the diagnostic performance of NI-RADS and to determine its potential role in guiding clinical decision-making and follow-up strategies.

## Supplementary Information

Below is the link to the electronic supplementary material.

Supplementary Information.

## Data Availability

Data are not publicly available due to privacy restrictions but can be requested from the corresponding author.

## Abbreviations

AUC: Area under the curve
BOLD: Blood oxygen level-dependent
DECT: Dual energy CT
ENE: Extranodal extension
HNSCC: Head and neck squamous cell carcinoma
MRI: Magnetic resonance imaging
NI: RADS-neck imaging reporting and data system
NPV: Negative predictive value
PET/CT: Positron emission tomography/computed tomography
PPV: Positive predictive value
ROC: Receiver operating characteristic
STIR: Short tau inversion recovery
