# Supplementary material for: Diagnostic performance of the NI-RADS system for detecting recurrence in head and neck squamous cell carcinoma: a monocentric pilot study
Source: J Cancer Res Clin Oncol. 2026 Jun 4;152(8):157. doi: 10.1007/s00432-026-06493-x (PMC13454081; doi:10.1007/s00432-026-06493-x)
Supplement: Supplementary file 1 [file 432_2026_6493_MOESM1_ESM.docx]

Fig. S1

Fig. S2
